# Blood Group Discrepancy in a Whole Blood Donor With Weak AB

**DOI:** 10.7759/cureus.40834

**Published:** 2023-06-22

**Authors:** Minal Wasnik, Saurabh Lahare, Peddinti Tejaswani

**Affiliations:** 1 Transfusion Medicine and Blood Bank, All India Institute of Medical Sciences, Raipur, IND

**Keywords:** discrepancy, anti-a1 antibody, a subgroup, ab blood group, weak subgroup

## Abstract

The ABO blood group system was the first to be discovered; still, there is an enigma in ABO subgroups. The A_1_ and A_2_ phenotypes account for about 99% of all A or AB blood group individuals, and the rest are the weaker subgroups. They usually are suspected in cases of blood group discrepancies. Meticulous serological testing with technical expertise will help to differentiate these subgroups. We describe a case of a healthy blood donor with blood group discrepancy due to a weak subgroup of 'A' in the AB blood group.

## Introduction

Karl Landsteiner, an Austrian physician, was the one who discovered the ABO blood group system in the 1900s. However, the ABO subgroups and other weaker variants create problems in blood grouping. ABO antigens are produced by the addition of a precursor oligosaccharide H chain with a terminal monosaccharide immunodominant sugar [1]. The weak expression of A, B, and H antigens on red blood cells can be either inherited or acquired. Although the weaker subgroups make up only 1%, yet, they pose a great challenge in routine immunohematology testing [2].

In the AB blood group, it is usually the A antigen that shows heterogeneity. It can be A1B, A2B, or other weaker subgroups. The strength of agglutination with anti-A, anti-B, anti‑AB, anti-H, and A_1_-lectin, presence or absence of ABO isoagglutinin in the serum, saliva secretor tests, and adsorption-elution, as well as molecular testing, are used to identify the weaker variations of the A antigen. Here, we report an incidental detection of weak 'A' antigen in the AB blood group in a young blood donor.

## Case presentation

A 30-year-old male came to our tertiary care teaching hospital's blood center as a replacement donor. He did not have any systemic illness, and there was no significant history. His hemoglobin was more than 12.5 gm%, and his weight was 65 kg. He was accepted for donation based on the screening criteria as per NBTC guidelines. We collected 450 ml of whole blood along with pilot tube samples.

Routine blood grouping from the pilot tube was performed by the conventional tube technique. The donor RBCs were tested with commercially available known anti-A, anti-B, anti-AB, and anti-D anti-sera (Tulip Diagnostics Pvt. Ltd.). For forward grouping, we prepared a 3-5% washed RBC saline suspension from the donor blood sample. Two drops of anti-A and anti-B and anti-AB were taken, and one drop of RBC suspension was added to labeled test tubes, respectively.

For reverse grouping, donor serum was tested against known RBCs. Two drops of the donor serum were taken, and one drop of in-house freshly prepared 3-5% saline suspension of pooled A-cells, B-cells, O-cells, and auto-control was added in labeled test tubes, respectively. The test tubes were centrifuged at 1000 rpm for 1 min, and the result was read against a well-lit background.

At room temperature, forward grouping showed anti-A:0, anti-B:4+, and anti-AB:4+ agglutination; however, reverse grouping showed 0 agglutinations with A-cells, B-cells, O-cells, and autocontrol. The forward grouping suggested blood group B, while the reverse grouping suggested blood group AB. Thus, there was a discrepancy in ABO forward and reverse blood grouping. The donor tested positive for anti-D. All the negative results were checked under the microscope. Testing with blood bag samples also showed similar results. The donor was called again and fresh blood and saliva sample was collected.

The fresh blood sample showed similar results at room temperature. The sample was tested with H and A1-lectin, which showed reactions as A1-lectin:0 and H-lectin:2+ at room temperature for 15 minutes. When incubated for 30 minutes at room temperature, the forward and reverse grouping results were the same. When incubated at 4ºC for 30 minutes, results were forward grouping: anti-A:0, anti-B:4+, anti-AB:4+, A1-lectin:0, and H-lectin:3+; reverse grouping: A-cells:2+, B-cells:0, O-cells:0, and autocontrol:0 (Table 1). The results were suggestive of cold-reacting low titer anti-A antibody, and we suspected anti-A1 antibody in the donor serum.

**Table 1:** Forward and reverse grouping of donor sample at room temperature and 4ºC RT: room temperature; min: minutes

	Forward					Reverse			
	Anti-A	Anti-B	Anti-AB	Anti-A_1 _lectin	Anti-H lectin	A_1_-cells	B-cells	O-cells	Auto-control
At RT	0	4+	4+	0	2+	0	0	0	0
RT 15 min	0	4+	4+	0	2+	0	0	0	0
RT 30 min	0	4+	4+	0	2+	0	0	0	0
4ºC 30 min	0	4+	4+	0	3+	2+	0	0	0

We further performed an adsorption elution test to resolve the discrepancy; 1 ml of donor red cells washed thrice was mixed with 1 ml of serum for known blood group B as it contains naturally occurring anti-A antibodies. It was incubated at 4ºC for 60 minutes and then centrifuged. The supernatant was removed. The red cells were washed with large quantities of cold saline, and the final wash was preserved. The heat elution method was used to recover the antibodies. The eluate, as well as the final wash, was tested in parallel with three O-cells and three A-cells at room temperature, 4ºC and 37ºC after incubating for 15 minutes. It showed 1+ agglutination with A-cells at 4ºC and 37ºC (Table 2). This suggested the presence of an 'A' antigen. Thus, reverse grouping showed reactivity with A-cells and eluate showed the presence of 'A' antigen; we concluded that he is having a subgroup of 'A' in blood group AB with the possibility of cold reacting anti-A_1_ antibody.

**Table 2:** Adsorption elution testing RT: room temperature

Eluate	37ºC	RT	4ºC
A-cells	1+	0	1+
O-cells	0	0	0
Final wash			
A-cells	0	0	0
O-cells	0	0	0

Inhibition testing was performed on a saliva sample. As there was unavailability of anti-Le^a^ antisera and known secretors, only normal saline was used as a control. His saliva showed the presence of A, B, and H antigens (Table 3).

**Table 3:** Saliva testing for secretor status

	Saliva+anti-A+A-cells	Saliva+anti-B+B-cells	Saliva+anti-H+O-cells	Normal saline+anti-A+A-cells	Normal saline+anti-B+B-cells	Normal saline+anti-H+O-cells
Reaction	0	0	0	1+	1+	1+

There was a dilemma in correctly labeling the subgroup. Based on the results of adsorption elution and secretor studies, we concluded that it was a weak 'A' in the AB blood group, and the possibility of Am or Ay was considered. Weak A subgroups sometimes show the presence of naturally occurring anti-A1 antibodies, which further helps in classifying it as a weak 'A' phenotype. In our case, we found a 2+ reaction at 4ºC with anti-A1 cells, which we considered as anti-A1 antibodies. In view of these immunohematological findings, in a 30-year-old young healthy male with no underlying illness or under treatment, we reported it as a weak 'A' subgroup in the AB blood group, Rh positive along with the presence of anti-A1 antibody. Further serum enzyme and molecular testing would have helped in confirming our findings; however, it could not be performed due to the lack of facilities.

## Discussion

The ABO blood group system was the first system to be discovered, but the occurrence of its weaker variants still poses an enigma for immunohematologists. The ABO gene is situated on the long arm of chromosome 9 (9q34) with seven exons. It has been studied that ABO gene mutations affect A and B glycosyltransferase enzyme activity and are responsible for ABO subtypes [3].

The variants of the 'A' antigen can be seen in both the blood group A and AB and are more common than the 'B' antigen variants. A1 and A2 form 99% of 'A' antigen phenotypes [4]. The rest are weaker variants, like Ax, A3, Aend, etc., including others that are difficult to type serologically. The weaker variants usually require advanced testing like serum transferase enzyme studies and 'A' substance quantification and molecular analysis for differentiating them accurately.

The weaker variants are usually suspected when a blood group discrepancy is encountered. We encountered a Type I blood group discrepancy, i.e., weak or missing antibodies in the reverse grouping. Sahu et al. reported 15 (0.12%) ABO blood group discrepancies among 12,715 samples studied. Out of the 15 discrepancies, six (40%) were attributed to weak or missing antibodies due to subgroups [5]. Extremes of age, immunodeficiency conditions, and other clinical conditions like post-hematopoietic stem cell transplant are a few possible causes of weak or missing reactivity of antibodies in the reverse grouping [2]. ABO subgroups also lead to Type I discrepancy. In our case, the donor was a healthy young male; thus, we ruled out other causes and considered the ABO subgroup.

Additionally, Das et al. studied 67,954 samples, out of which 6618 were AB blood group. Among the AB blood group, 84.93% were A1B, 15.05% A2B, and only 0.015% were weak subgroups of AB. They mentioned that subgroups are mainly of academic interest and group 'A' phenotypes which show weaker serologic reactivity than A2 are considered as weak subgroups [6].

Cartron demonstrated that there is a gradual decrease in the standard agglutinability of weak 'A' RBC with human anti-A (B) sera, from A3 red cells (63% ± 10%) to Ax (33% ± 10%), Aend (10% ± 5%) then Am, Ay, and Ael (0%). The direct measurement of A antigen site densities was also performed, and the mean values were 35,000 A sites/RBC in A3, 4800 in Ax, 3500 in Aend, and 700 ‘A’ antigen sites/RBC in Am and Ael respectively [7].

Several serological tests which include reactivity of red cells with anti-A, anti-AB, and anti-H followed by secretor study and adsorption elution tests can be used to identify weak phenotypes such as A_3_, A_x_, A_end_, A_m_, A_y_, and A_el_ [2]. We performed these investigations as per the standard operating procedures of our department based on standard guidelines described elsewhere [8]. We concluded that it was a weak ‘A’ subgroup in AB, and the possibility of A_m_ or A_y_ was considered. In the case of A_y_, the eluate shows a weaker reaction to A-cells as compared to A_m_. Our case also showed a 1+ reaction on adsorption elution.

Das et al. reported a case of weak ‘A’ subgroup in a healthy female donor detected due to blood group discrepancy. The authors pointed out that serologically A_y_ is almost similar to A_m_ and even the adsorption elution test fails to differentiate the two phenotypes. Thus, they reported it as a weak ‘A’ subgroup [9].

It has been observed that weak ‘A’ subgroups show the presence of naturally occurring anti-A_1_ antibodies, which further helps in classifying it as a weak ‘A’ phenotype. We found 2+ reactions at 4ºC with anti-A_1_ cells, which most likely could be anti-A_1_ antibodies. Thus, we labeled our case as weak ‘A’ in AB, Rh positive with anti-A_1_ antibodies. For confirmation, tests like serum transferase levels and molecular genetic testing should be used. This case points out the importance of performing meticulous serological testing in cases of blood group discrepancies, even if there is a lack of genetic testing.

Although we highlighted the importance of meticulous serological testing in a case of blood group discrepancy, the lack of facilities to perform confirmatory tests like serum transferase levels, ‘A’ substance, and molecular genotyping was the limiting factor.

## Conclusions

Our case shows that weak ‘A’ subgroups can be missed out or mistyped especially in the AB blood group as they are rare. This could lead to delayed hemolytic transfusion reaction and reduced cell survival post-transfusion. It is advised that forward and reverse grouping should always be performed; if any discrepancy is encountered, meticulous serological testing should be performed to resolve it. With serological testing alone, it is possible to differentiate weak subgroups of ‘A’ to a great extent. In developing countries like India, blood centers should establish procedures to detect and differentiate weak subgroups and ensure safe blood transfusion and transplantation.
